# Repurposing an antidandruff agent to treating cancer: zinc pyrithione inhibits tumor growth *via* targeting proteasome-associated deubiquitinases

**DOI:** 10.18632/oncotarget.14572

**Published:** 2017-01-10

**Authors:** Chong Zhao, Xin Chen, Changshan Yang, Dan Zang, Xiaoying Lan, Siyan Liao, Peiquan Zhang, Jinjie Wu, Xiaofen Li, Ningning Liu, Yuning Liao, Hongbiao Huang, Xianping Shi, Lili Jiang, Xiuhua Liu, Q. Dou Ping, Xuejun Wang, Jinbao Liu

**Affiliations:** ^1^ State Key Laboratory of Respiratory Disease, Protein Modification and Degradation Laboratory, Department of Pathophysiology, Guangzhou Medical University, Guangdong 510182, China; ^2^ Department of Gastroenterology, Guangzhou Digestive Disease Center, Guangzhou First People's Hospital, Guangzhou Medical University, Guangzhou 510180, China; ^3^ Guangzhou Research Institute of Cardiovascular Disease, The Second Affiliated Hospital, Guangzhou Medical University, Guangzhou, Guangdong 510260, China; ^4^ Institute of Environmental and Analytical Sciences, College of Chemistry and Chemical Engineering, Henan University, Kaifeng, Henan 475004, China; ^5^ The Molecular Therapeutics Program, Barbara Ann Karmanos Cancer Institute, and Departments of Oncology, Pharmacology and Pathology, School of Medicine, Wayne State University, Detroit, Michigan 48201-2013, USA; ^6^ Division of Basic Biomedical Sciences, Sanford School of Medicine of the University of South Dakota, Vermillion, South Dakota 57069, USA

**Keywords:** zinc pyrithione, proteasome, deubiquitinases, DNA damage, tumor

## Abstract

The ubiquitin-proteasome system (UPS) plays a central role in various cellular processes through selectively degrading proteins involved in critical cellular functions. Targeting UPS has been validated as a novel strategy for treating human cancer, as inhibitors of the 20S proteasome catalytic activity are currently in clinical use for treatment of multiple myeloma and other cancers, and the deubiquitinase activity associated with the proteasome is also a valid target for anticancer agents. Recent studies suggested that zinc pyrithione, an FDA-approved antidandruff agent, may have antitumor activity, but the detailed molecular mechanisms remain unclear. Here we report that zinc pyrithione (ZnPT) targets the proteasome-associated DUBs (USP14 and UCHL5) and inhibits their activities, resulting in a rapid accumulation of protein-ubiquitin conjugates, but without inhibiting the proteolytic activities of 20S proteasomes. Furthermore, ZnPT exhibits cytotoxic effects against various cancer cell lines *in vitro*, selectively kills bone marrow cells from leukemia patients *ex vivo*, and efficiently inhibits the growth of lung adenocarcinoma cancer cell xenografts in nude mice. This study has identified zinc pyrithione, an FDA-approved pharmacological agent with potential antitumor properties as a proteasomal DUB inhibitor.

## INTRODUCTION

Ubiquitin-mediated proteasomal proteolysis is the major mechanism of intracellular protein degradation responsible for the removal of defective and misfolded polypeptides in human cells, and plays a crucial role in the regulation of numerous cellular and physiologic functions [1]. Abnormalities in the ubiquitin-mediated processes have been linked to many human diseases, including neurological disorders, viral infection, and cancer [2–4].

In the past decade, the ubiquitin-proteasome system (UPS) has attracted increasing attention as a target for novel cancer therapeutics. The approval of the proteasome inhibitor Velcade (bortezomib) for the treatment of multiple myeloma and mantle cell lymphoma has validated UPS as a valid target for cancer treatment [5, 6]. Unfortunately, like most of the other chemotherapeutics, 20S proteasome inhibitors such as bortezomib suffer from a narrow therapeutic index, since the proteasome activity is essential to many cellular pathways of all cells [7]. One way to overcome this shortcoming of 20S proteasome inhibitors is to target the upstream of UPS i.e., enzymes responsible for the ubiquitin conjugation and deconjugation, in order to improve target specificity and decrease toxicity.

Deubiquitinases (DUBs) are a class of enzymes that catalyze the hydrolysis of the isopeptidyl bond formed between ubiquitin and the target protein and regenerate free ubiquitin monomers [8]. Ubiquitin is removed from substrate proteins prior to proteasomal degradation *via* the action of DUBs. The human genome encodes roughly 100 DUBs. DUBs can be classified into five families and there are three proteasome-associated DUBs, USP14 and UCHL5, which are cysteine proteases, and a metalloprotease RPN11 [7, 8]. The relationship between these proteasomal DUBs and their physiological roles in regulating substrate degradation are complex and not yet completely understood. Many of DUBs have been identified as oncogenes or tumor suppressors due to their regulatory functions on the homeostasis of other proteins involved in tumor development. Recent reports have shown that DUBs are emerging as promising targets for pharmacological intervention [9–12]. The advantage of inhibiting DUBs, especially the proteasome-associated DUBs, lies in the potential specificity of therapeutic intervention that can lead to improved efficacy and side effects.

Zinc pyrithione (ZnPT; CAS# 13463-41-7), a coordination complex of zinc, has been widely used as an antimicrobial compound in antidandruff shampoos and in antifouling paints for over 50 years [13–15], and has also been approved by FDA as an therapeutic drug for topical treatment of UVB-induced epidermal hyperplasia [16]. Recent research suggests therapeutic potential of ZnPT for cancer intervention. It has been shown that ZnPT kills cancer cells *via* induction of zinc-dependent cell death [16]. However, more recent studies revealed that ZnCl_2_was far less toxic than ZnPT to AML cells, indicating that the antileukemic effects of ZnPT might not be mediated solely by inorganic Zn^2+^ions [17]. Moreover, earlier studies also suggest that ZnPT, like metal-based drug cisplatin/CDDP, induces a DNA-damage response followed by apoptosis [18]. The mechanism underlying such effects, however, remains poorly understood.

We have reported that metal-containing compounds could induce cytotoxicity in cancer cells by acting as the proteasome-associated DUBs’ inhibitors [19]. In the present study, we demonstrate that ZnPT blocks the deubiquitinase activity of the proteasome and induces a rapid accumulation of protein-ubiquitin conjugates, but has no inhibitory effect on the proteolytic activities of the 20S core particle (CP). Furthermore, ZnPT exhibits cytotoxic effects against various cancer cell lines *in vitro*, efficiently inhibits the growth of lung adenocarcinoma cancer cell xenografts in nude mice, and selectively kills bone marrow cells from AML patients *ex vivo*.

## RESULTS

### Induction of apoptosis in cancer cells by ZnPT

**Figure 1:**
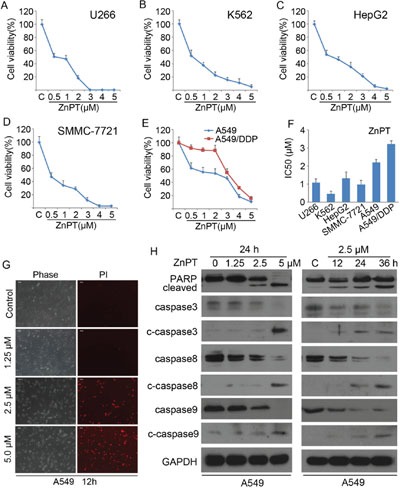
Induction of apoptosis in cancer cells by ZnPT **A-E**. Cytotoxic curves in multiple cancer cells. U266 (A), K562 (B), HepG2 (C), SMMC-7721 (D), A549 (E) and A549/DDP (E) cells were treated with escalating doses of ZnPT for 48 hours, cell viability was detected by MTS assay. n=3, Mean±SD. **F**. Viable cells by 50% compared with vehicle control (IC50) in a panel of cancer cell lines treated with ZnPT for 48 hours. **G**. ZnPT induces cell death in A549 cells. A549 cells were treated with the indicated doses of ZnPT, then PI was added to the cultured cells after 12 hours treatment, PI-positive staining was monitored under an inverted microscopy and typical images were shown. **H**. ZnPT induces cleavage of PARP, caspase-3, -8, -9 in A549 cells. A549 cells were treated with ZnPT at various doses or for various times, PARP, and caspase-3, -8, -9 cleavage were analyzed by immunoblotting. GAPDH was detected as a loading control.

To investigate cytotoxic effects of ZnPT on cancer cells, a panel of cancer cell lines, including U266, K562, HepG2, SMMC-7721, A549 and A549/DDP, were assayed using MTS. After a period of 48 hours treatment with escalating concentrations of ZnPT, cell viability was determined for all these 6 cell lines. We observed that ZnPT decreased cell viability in a dose-dependent fashion in all the tested cancer cells, including those resistant to Cisplatin (CDDP) (Figure 1A-1E). ZnPT displayed sub- or low-micromolar IC_50_ values (< or =2μM) against all of cancer cell lines except A549/DDP cells (Figure 1F). When compared with the parental A549 cells, A549/DDP cells are less sensitive to ZnPT at dose < 3 μM (IC_50_ 3.3 *vs*. 2.2 μM; Figure 1F); however, at 3.0μM or higher concentrations, they are as sensitive to ZnPT as A549 cells (Figure 1E).

We next tested the ability of ZnPT to induce cell death in cisplatin-sensitive cell line. A549 cells were exposed to escalating doses of ZnPT for 12 hours, followed by recording the PI (propidium iodide)-positive cells with fluorescence microscopy. ZnPT was found to induce significant A549 cell death in a dose-dependent manner (Figure 1G). To analyze whether the ZnPT-induced cytotoxicity of cancer cells is associated with caspase activation, the processing of the caspase cascade was investigated. A549 were incubated with the different doses of ZnPT for indicated hours, followed by Western blot analysis (Figure 1H). The results showed that the induction of PARP cleavage by ZnPT treatment paralleled the increase of the active forms of caspases-3, -8, -9 in a dose- and time-dependent pattern. Collectively, these results indicate that ZnPT triggers caspase-dependent apoptosis in lung adenocarcinoma cancer cells.

### Rapid accumulation of ubiquitin conjugates in cancer cells treated with ZnPT

**Figure 2:**
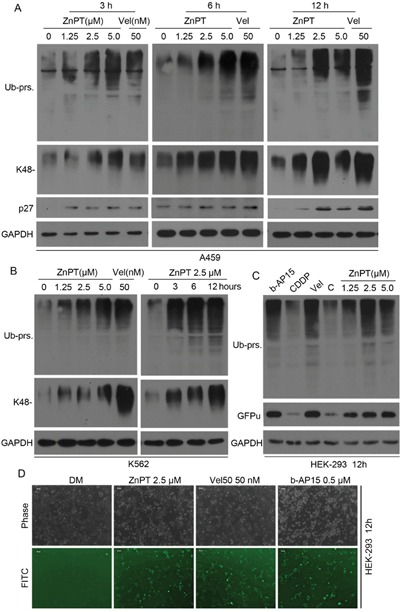
Rapid accumulation of ubiquitin conjugates in cancer cells treated with ZnPT **A, B**. Dose- and time-dependent accumulation of proteasomal substrates in cancer cells.A549 (A) were treated with ZnPT(1.25, 2.5, 5 μM) and Velcade (Vel, 50 nM) for 3, 6, 12hours respectively; K562 cells were treated with escalating ZnPT for 12hours (B, left) or 2.5μM ZnPT for indicated times (B, right), followed by detecting the protein levels of ubiquitin conjugates (totalUb-prs andK48-linked Ub-prs) and p27 with western blot analysis. **C, D**. The accumulation of fluorescent proteasome reporter GFPu. HEK-293 cells stably expressing GFPu were treated with ZnPT for 12 hours, Ub-prs and GFPu protein were detected by Western blot (C) or imaged (D) under an inverted fluorescence microscope (scale bar, 50μm). Vel and b-AP15 were used as positive controls.

Previously, we have reported that CuPT could induce cancer cell apoptosis by directly inhibiting cellular proteasome function [19]. To study the effect of ZnPT on the UPS as a pyrithione complex, we first measured the levels of ubiquitinated proteins in A549 and K562 cells incubated with ZnPT and Velcade. As expected, ZnPT induced dramatic increase in total ubiquitinated proteins (Ub-prs) and K48-linked Ub-prs as well as proteasome substrate p27 in a dose- and time- dependent fashion (Figure 2A and 2B). Besides endogenous substrates, ZnPT also accumulated a surrogate proteasome substrate (GFPu) and Ub-prs in a stable GFPu-HEK293 cell line (Figure 2C and 2D). Surprisingly, ZnPT at 2.5μM induced similar high levels of GFPu as Velcade and b-AP15 used at the indicated doses in these cells. Velcade is a classical 20S proteasome inhibitor, and b-AP15 is a reported inhibitor of proteasomal DUBs (Figure 2C and 2D). Instead, CDDP failed to inhibit proteasome function, even though at much higher doses compared with ZnPT (Figure 2C). These results suggest that ZnPT, similar to velcade and b-AP15, can induce proteasome inhibition.

### ZnPT inhibits the UPS by targeting 26S-associated DUBs USP14 and UCHL5

**Figure 3:**
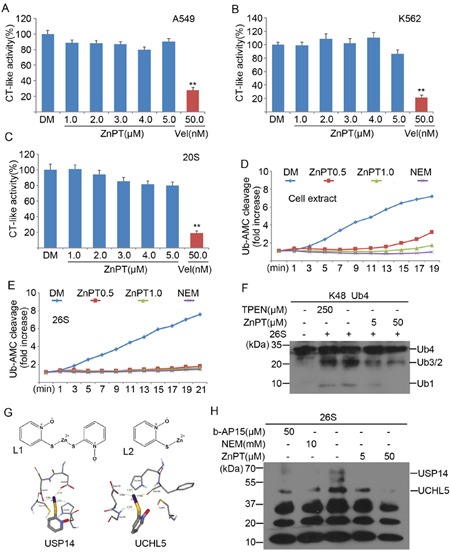
ZnPT inhibits the UPS by targeting 26S-associated DUBs USP14 and UCHL5 **A-C**. The *in situ* and *in vitro* effects of ZnPT on proteasome peptidase activity. Lysates from ZnPT or Vel-treated A549 (A) and K562 (B) cells were analyzed for proteasome chymotrypin-like activity. Purified 20S proteasomes (C) were treated with indicated doses of ZnPT, chymotrypin-like activity was measured using specific synthetic fluorogenic substrates. Vel was used as a positive control. Values are expressed as mean ± SD (*n* = 3). **P < 0.01, compared with each control. **D, E**. The effect of ZnPT on DUB activities. A549 celllysates (D) and purified 26S proteasomes (E) were exposed to ZnPT (0.5, 1.0 μM) and dynamic DUB activity was measured. NEM was used as a positive control. **F**. Inhibition of ubiquitin chain disassembly by ZnPT. Purified 26S proteasomes were treated with ZnPT (5.0, 50μM), TPEN (250 μM), or b-AP15 (50 μM), followed by the detection of disassembly of K48-linked ubiquitin tetramers using western blots. **G**. Computational molecular docking of ZnPT with UCHL5 and USP14. The following data were shown: the structure of zinc pyrithione (L1); the structure of zinc pyrithione intermediate (L2); the binding modes of compound L2 at the active site of USP14and UCHL5. **H**. Abolishment of Ub-VS labeling of proteasomal DUBs by ZnPT. Purified 26S proteasomes were treated with ZnPT (5.0, 50μM), NEM (10 mM), or b-AP15 (50 μM), followed by labeling with HA-UbVS and immunoblottingusing an anti-HA antibody.

To determine the molecular mechanism for ZnPT-induced proteasome inhibition, we first tested the direct effect of ZnPT on proteasome peptidase activities *in vitro* or in live K562 and A549 cells. It was found that ZnPT showed no significant effects on proteasome CT-like activities in either purified human 20S proteasome or cultured cells, whereas Velcade exhibited substantially inhibitory effect in all assays (Figure 3A-3C). These results suggest that ZnPT does not directly block the 20S proteasome peptidase activity, which is consistent with the previous findings that zinc complex inhibits the UPS independently of the 20S [27]. We next assessed the possibility of DUB inhibition by ZnPT. As shown in Figure 3D, distinctive reduction of cellular DUB activity was detected using Ub-AMC as a substrate in K562 cells following ZnPT treatment. As a positive control, N-ethylmaleimide (NEM) completely suppressed cellular DUB activity. Similar to NEM, ZnPT at 0.5μM could significantly inhibit the purified 26S proteasome-associated DUB activity (Figure 3E). This effect was further confirmed by *in vitro* disassembly of purified tetraubiquitin chains (Ub4). Figure 3F shows that ZnPT could dose-dependently block 26S-mediated K48-linked tetra-Ub4 chain disassembly *in vitro*. These tests suggest that ZnPT inhibits 26S proteasome-associated DUB activity. Then we performed a computational docking study to predict the binding information between ZnPT and 26S-associated DUBs including USP14 and UCHL5. The chemical structures of ZnPT (L1) and its hydrolysate product (L2) are illustrated in Figure 3G (upper). In our docking results, ZnPT was found to strongly bind to the active sites of the two DUBs, USP14 and UCHL5. As displayed in Figure 3G, in the binding site of USP14, the Zn^2+^ can form three coordination bonds with catalytic core, with distances of 3.319, 2.197 and 2.302Å, respectively. Similarly, in the binding site of UCHL5, there are three coordination bonds between the Zn^2+^ and catalytic core, with corresponding bond lengths of 2.321, 2.164 and 2.482 Å. In addition, one hydrogen bond of 2.372 Å is formed between the S atom of ZnPT and the polar H of Gly180 (Figure 3G lower). These computational results indicate that ZnPT can bind to the catalytic cores of USP14 and UCHL5 through coordination bonds and hydrogen bonds. Competitive labeling experiments using HA-tagged ubiquitin vinylsulphonone (HA-UbVS) revealed results consistent with this computational hypothesis. As an active site probe of cysteine DUBs, HA-UbVS can bind covalently to the active site of USP14 and UCHL5. Incubation of ZnPT at 5μM with 26S proteasomes substantially reduced UbVS binding to UCHL5 and abolished UbVS binding to USP14, and when ZnPt is increased to 50μM, UbVS binding to both UCHL5 and USP14 was abolished (Figure 3H). These computational and experimental results demonstrate that ZnPT can target two proteasome-associated DUBs, UCHL5 and USP14.

### ZnPTdoes not directly bind DNA nor induce DNA damage *in vitro*

**Figure 4:**
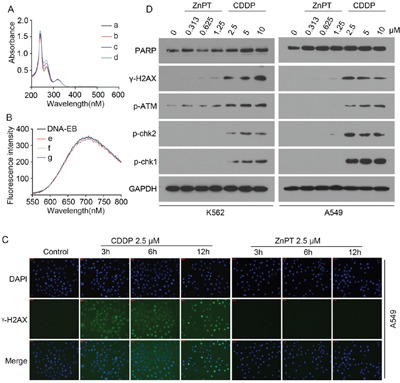
ZnPT does not directly bind DNA or induce DNA damage *in vitro* **A**. UV absorption spectra of ZnPT with indicated concentrations of DNA. c(ZnPT)=15.2 μM, c(ZnPT)= [0(a), 14.2(b), 28.5(c), 42.7(d)μM]. **B**. Fluorescence spectra of DNA-EB with indicated concentrations of ZnPT. c(EB)=2μM; c(DNA)=14μM, c(ZnPT)=[12(e), 31(f), 64(g) μM]. **C**. γ-H2AX analysis at different times after CDDP or ZnPT treatment. A549 cells weretreated with CDDP (2.5 μM) or ZnPT(2.5 μM) for indicated time points, followed by immunofluorescence staining assay. Green: γ-H2AX, blue: nuclei stained with DAPI (scale bar, 50μm). **D**. Immunoblotting for the detection of DNA damage pathway-related proteins. A549 and K562 were incubated with indicated concentrations of ZnPT or CDDP for 12 hours, DNA damage-related proteins including γ-H2AX, p-ATM, p-chk2, p-chk1 and PARP were detected by Western blot.

Cisplatin is a prevailing paradigm for the use of metal medicine and exerts its cytotoxicity by formation of covalent DNA adducts. To confirm whether ZnPT could bind DNA duplex as a metal complex, we employed UV absorption spectroscopy and fluorescence spectroscopy, using ethidiumbromide (EB) as a fluorescence probe of DNA. Figure 4A shows the UV absorption spectra of ZnPT with increasing concentration of calf thymus DNA. With the addition of DNA, there was no shift in the ZnPT's characteristic absorption peak at 230 nm. The fluorescence emission spectra of EB bound DNA in the absence and the presence of ZnPT is given in Figure 4B. With increasing the concentration of ZnPT, the fluorescence intensity of the system did not show notable changes in the wavelength of maximum emission. These results indicate that ZnPT does not directly bind to DNA. To study whether ZnPT could induce DNA damage *in vitro*, phosphorylation of H2AX at serine 139 (γ-H2AX) was used as a hallmark of DNA damage response. We monitored the γ-H2AX level of A549 cells treated with CDDP (2.5 μM) or ZnPT(2.5 μM) using immunofluorescence. The result showed typical time-dependent formation of γ-H2AX foci after treatment of CDDP, but not of ZnPT (Figure 4C). Then we investigated the effect of ZnPT on the DNA damage response pathway. It was found that CDDP, but not ZnPT, induced the expression of γ-H2AX and DNA damage signaling proteins including p-ATM, p-chk2, and p-chk1, in a dose-dependent manner (Figure 4D). These results indicate that ZnPT, at a concentration sufficient to inhibit proteasomal DUBs and cell growth, does not remarkably cause DNA damage responses.

### Proteasome inhibition is required for ZnPT to induce apoptosis

**Figure 5:**
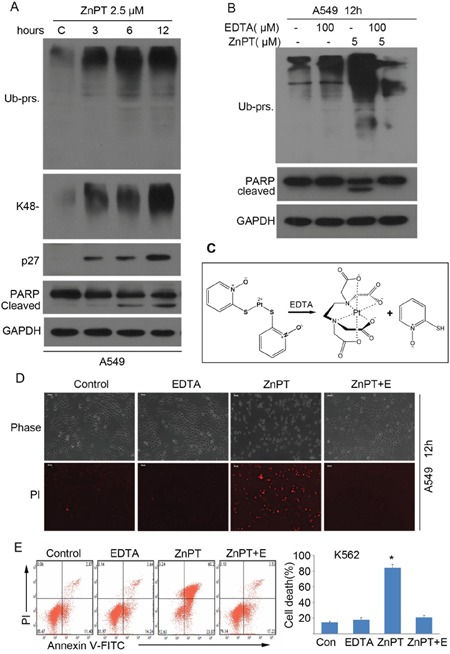
Proteasome inhibition is required for ZnPT to induce apoptosis **A**. ZnPTinduces proteasome function inhibition and apoptosis at different time course. A549 cells were treated with ZnPT (2.5 μM), then the cells were collected at the indicated time points for western blot analysis for ubiquitinated proteins including total Ub-prs and K48-linked Ub-prs, p27 and PARP cleavage. **B**. EDTA effectively prevents ZnPT-induced proteasome inhibition and apoptosis. A549 cells were treated with ZnPT in the presence/absence of chelating agent EDTA (100 μM) for 12 hours, total Ub-prs and PARP cleavage were detected by Western blot. **C**. An illustration of the coordination of Zn^2+^ with ethylenediaminetetraacetic acid (EDTA) from ZnPT. **D, E**. EDTA completely reversed ZnPT-induced cell death. A549 cells were treated with ZnPT, alone or in combination with chelating agent EDTA (100 μM) for 12 hours, followed by imaging under fluorescence microscopy (D) or flow cytometry (E) for recording death cells. Values are expressed as mean ± SD (*n* = 3). *P < 0.05, compared with each control.

To determine whether the inhibition of proteasomes is responsible for induction of apoptosis, we first tested if the proteasome inhibition occurs prior to cell apoptosis. K562 cells were incubated with either vehicle or 2.5μM of ZnPT for 3, 6, 12 hours, followed by detection of total, K48-linked ubiquitinated proteins and proteasome substrate p27 using Western blot analyses. We observed an accumulation of ubiquitinated proteins during the course of ZnPT treatment as early as 3hours (Figure 5A). However, PARP cleavage, an apoptosis indicator, was not discernible until 6 hours after ZnPT treatment (Figure 5A). These results suggest that the ZnPT-induced apoptosis occurs after the proteasome inhibition. Next, we tested the effect of ethylenediaminetetraacetic acid (EDTA), a powerful chelating agent for metal ions, on the ZnPT-induced proteasome inhibition and cell death. As expected, EDTA completely inhibited ZnPT-induced ubiquitinated protein accumulation and, PARP cleavage in A549 cells (Figure 5B). Figure 5C illustrates that ZnPT could be inactivated by EDTA by chelating Zn^2+^ from ZnPT. This abolished effect on cell death was also confirmed using propidium iodide (PI)-staining in A549 cells followed by inverted fluorescent microscopy (Figure 5D), and using flow cytometry in K562 cells with Annexin V/PI staining (Figure 5D). Therefore, proteasome inhibition is required for apoptosis induction by ZnPT.

### ZnPT induces cytotoxicity and proteasome inhibition in cancer cells from leukemia patients

**Figure 6:**
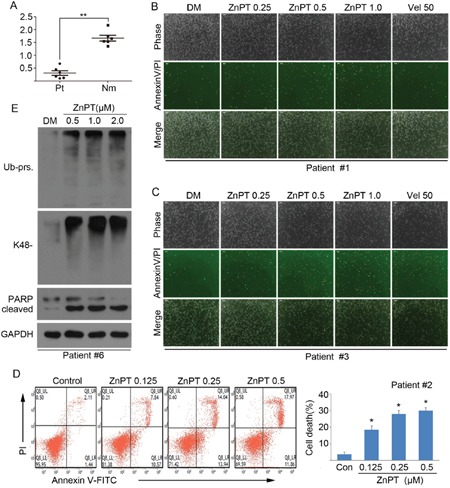
Effect of ZnPT on primary monocytes from leukemia patients **A**. Bone marrow cells from 6 leukemia patients (Pt) and peripheral blood mononuclear cells from 6 healthy volunteers (Nm) were treated with ZnPT at the indicated doses for 48 hours, then the cell viability was detected by MTS assay. The scatter plots of the IC_50_ values of ZnPT in each group were shown. Values are expressed as mean ± SD (*n* = 3). **P < 0.01, compared with Pt group. **B-D**. Cancer cells from leukemia patients were treated with increasing doses of ZnPT for 24 hours, cell apoptosis from two samples (#1 and #3) were recorded with AnnexinV/PI double staining under a fluorescent microscopy (B, C) or one sample (#2) was analyzed by flow cytometry (D). Values are expressed as mean ± SD (*n* = 3). *P < 0.05, compared with each control. **E**. Leukemia cells were incubated with ZnPT at the indicated doses for 24 hours, ubiquitinated proteins and PARP were detected by western blot. Representative images from one sample (#6) were shown.

To evaluate the possible antineoplastic action of ZnPT at the preclinical level, we test the *ex vivo* cytotoxicity of ZnPT on the bone marrow cells from six leukemia patients. Peripheral blood mononuclear cells (PBMCs) from six healthy individuals were used as controls. It was found that the average IC_50_ values in normal PBMCs after ZnPT exposure for 48 hours were1.623±0.122 μM, over 5-fold higher than that for primary monocytes from leukemia patients (0.308±0.097μM) (Figure 6A). Next, we applied the flowcytometry and fluorescence microscopy to detect the ZnPT-induced apoptosis using Annexin V/PI staining in the monocytes from leukemia patients, Figure 6B and 6C exhibits results of dose-dependent apoptosis in response to ZnPT, and the consistent results were seen in the Figure 6D. Also, treatment with ZnPT significantly induced accumulation of ubiquitinated protein and increased levels of cleaved PARP in cancer cells from AML patients (Figure 6E). These results demonstrate that ZnPT has a therapeutic effect on leukemia patients and induces *ex vivo* proteasome inhibition.

### ZnPT inhibits proteasome function and tumor growth *in vivo*

**Figure 7:**
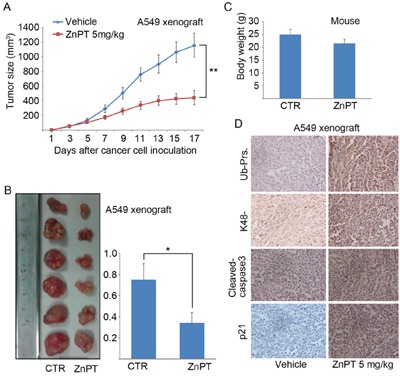
ZnPT inhibits tumor growth and proteasome function *in vivo* **A-B**. A549 (1×10^6^) cells were subcutaneously injected into BALB/c nude mice. Mice bearing A549 xenograft were treated with vehicle or ZnPT (5mg/kg/day, *i.p*.) for 15 days. Tumor size and body weight were recorded every other day. Tumor size (A), tumor images (B), tumor weight (B) and body weight C. were shown. n=6, mean±SD.*P < 0.05, **P < 0.001, compared with each control. **D**. Immunohistochemical staining results of total Ub-prs or K48-linked Ub-prs, proteasome substrate protein (p21) and cleaved caspase-3 in A549 cell xenografts from nude mice treated with vehicle or ZnPT. Representative images are shown (200x).

To further confirm the preclinical antineoplastic action of ZnPT, immunodeficient BALB/c nude mice were injected subcutaneously with A549 cells, followed by treatment with intraperitoneal injections of ZnPT. At doses that did not cause any significant body weight reduction, ZnPT strongly reduced the growth of A549-derived xenografts as shown in the tumor size curves and images (Figure 7A and 7B). ZnPT-treated mice also showed a significant reduction in tumor weight (Figure 7B), consistent with the tumor size data. Importantly, the body weight was relatively stable in ZnPT-treated group (Figure 7C). These results indicate that ZnPT could selectively inhibit *in vivo* tumor growth without apparent toxicity to host animals. We previously found that one of the effects of ZnPT treatment *in vitro* is the proteasome inhibition (Figure 2A and 2B). Consistent with these data, highly increased levels of typical proteasome substrates including total and K48-linked ubiquitinated proteins and p21, accompanied by caspase-3 activation, were observed in ZnPT-treated tumor xenografts with immunohistochemistry staining analysis (Figure 7D), suggesting that proteasome is a molecular target of ZnPT *in vivo*.

## DISCUSSION

ZnPT is an important FDA-approved microbicidal OTC drug and with FDA-regulated applications in skin products. Topical safety and toxicity profile of ZnPT have been studied to some extent previously [21]. Recently, anticancer effects of ZnPT have been described in leukemia and oral cancer cells [17, 22], but the specific mechanism underlyingits biological activities remains poorly understood.

In the past decade, Dou and his colleagues designed many metal compounds to target the 26S proteasome. These include, butare not limited to, copper-, zinc- and gold-containing complexes which have made targeting the UPS with metal-based compounds as an emerging concept in development of novel anticancer drugs [23–26].

Given that the discovery and development of new drugs is a very lengthy and costly process, re-investigating existing drugs for new therapeutic use has emerged as an attractive strategy in cancer drug development [27]. For this purpose, we substantiated, for the first time, ZnPT-mediated anticancer effects by inhibition of 26S proteasome-associated DUBs, demonstrated its mechanism of action on cancer cells *in vitro* and *in vivo*. The aim of this study was to identify clinically used antidandruff agent that will serve as an effective alternative to current chemotherapeutic agents for cancer therapy. Based on our findings, ZnPT treatment efficiently suppressed the cell viability of multiple types of cultured cancer cells, especially the cisplatin-resistant cell lines, suggesting that ZnPT could be used in cisplatin-resistant tumors. In addition to the established cancer cell lines, the IC_50_ values of ZnPT for primary cancer cells from leukemia patients were much lower than those for normal mononuclear cells. Of note, ZnPT significantly retarded tumor growth at a well-tolerated dose *in vivo*. Our results show that ZnPT is a promising anticancer drug candidate with potential clinical significance.

ZnPT has been reported to target cancer cells by increasing intracellular Zn^2+^ concentration, followed by ERK activation, reactive oxygen species generation [28], loss of NF-κB expression and its target genes in AML cells [17], activation of p53 and its dependent genes Puma and Bax associated with loss of mitochondrial membrane potential [29]. Therefore the detailed mechanism of ZnPT-induced apoptosis in cancer cells was not clear. Our previous report found that CuPT, a cooper and PT-chelating product, interfered with the UPS *via* inhibiting DUBs [19]. Similar to CuPT, ZnPT induced marked accumulation of exogenous and endogenous proteasome substrates *in vitro*, *in vivo* and *ex vivo*. We have found that ZnPT-induced Ub-prs and GFPu accumulation is comparable to that induced by bortezomib but the mediating mechanism is distinct to that of bortezomib. The computational docking calculations and enzyme inhibition assays show that ZnPT displays properties to competitively inhibit 26S proteasome-associated DUB activities (USP14 and UCHL5). Since we have identified that the bio-target of ZnPT involves USP14 and UCHL5, whether overexpression of USP14 or UCHL5 could add resistance of cancer cells to ZnPT should be investigated in the future.

Notably, ZnPT was shown to induce DNA-damage responses in primary human skin keratinocytes and melanocytes [18]. In our study, however, ZnPT treatment in cultured cancer cells did not induces DNA-damage responses, as reflected by the absence of γ–H2AX foci formation and failure to increase DNA-damage response-linked phosphorylation of ATM, H2AX, Chk1 and Chk2. This is consistent with the reported findings that ZnPT targets do not involve DNA [17]. Accordingly, no direct binding between ZnPT and calf thymus DNA was observed under simulated physiological conditions (Figure 4A-4B), which further rules out the possibility that the anticancer properties of ZnPT might be mediated by DNA damage.

Previous studies have indicated that blockade of tumor proteasome activity results in cancer cell apoptosis [30]. As expected, we found that cleaved PARP (an apoptosis indicator) occurs after proteasome inhibition in ZnPt-treated cells. Importantly, Chelating agent EDTA inhibits both ZnPT-induced proteasome inhibition and apoptosis. Taken together, proteasome inhibition by ZnPT contributes to its induced apoptosis. It is possible that proteasome inhibition by ZnPT may result in increased ER stress, which subsequently accumulates Ca^2+^ in cytoplasm to cause permeabilization of the mitochondrial outer membrane, followed by cytochrome c and AIF release, leading to caspase activation.

In conclusion, we found that ZnPT could inhibit 26S proteasome-associated DUBs but not the 20S proteasome. Similar to CuPT, some other non-proteasomal DUB in the cytoplasm was inhibited by ZnPT as well, which needs to be further explored in the future study. The *in vitro* studies provided in depth understanding of its mechanism of action in cancer cells. The pre-clinical results from the *in vivo* mouse xenograft cancer model and the *ex vivo* cells from leukemia patients point to ZnPT as a promising candidate in treatment of cancer. This drug could be clinically evaluated and moved into the clinic as it is already FDA approved.

## MATERIALS AND METHODS

### Cell culture

U266 (myeloma cells), K562 (chronic myelogenous leukemia), HepG2 (liver hepatocellular carcinoma), SMMC-7721(Human hepatoma cells) and HEK293 (human embryonic epithelial cells) were obtained from American Type Culture Collection (Manassas, VA, USA), A549and cisplatin-resistant A549/DDP (human lung adenocarcinoma) was a gift of Dr. Z. He. Most of cell lines were cultured in RPMI 1640(Gibco-Invitrogen, Carlsbad, CA, USA) containing 10% fetal bovine serum (Gibco-Invitrogen, Carlsbad, CA, USA), 100 units/mL of penicillin and 100 g/mL of streptomycin.A549/DDP cells were routinely maintained in the same medium but supplemented with 1.5 μg/ml cisplatin, which was removed before experiments were started by a wash-out period of 2 to 3 days. HEK293 cultured in DMEM medium with high glucose containing 10% fetal bovine serum (HyClone, Logan, UT, USA). HEK-293 cells stably expressing GFPu were created as we previously described [31]. GFPu is a proven specific surrogate substrate of the UPS [32].

### Reagents and antibodies

ZnPT, cisplatin and b-AP15 were purchased from Sigma-Aldrich Inc. (St. Louis, MO, USA); human 20S and 26S proteasomes, Suc-Leu-Leu-Val-Tyr-aminomethylcoumarin (Suc-LLVY-AMC), HA-Ubiquitin-Vinyl Sulfone (HA-Ub-VS), K48-linked tetra-ubiquitin and ubiquitin-AMC were purchased from Boston Biochem (Cambridge, MA, USA); bortezomib was purchased from BD Biosciences (San Jose, CA, USA). The sources of antibodies used in this study: anti-ubiquitin (P4D1), anti-p27 (F-8), anti-GFP (B-2) were from Santa Cruz Biotechnology (Santa Cruz, CA, USA); anti-caspase3 (8G10), anti-caspase8 (1C12), anti-caspase9 (C9), anti-PARP, anti-p21 Waf1/Cip1 (DCS60), anti-K48-linkage specific polyubiquitin (D9D5), anti-phospho-histone H2AX (Ser139) (20E3), anti-phospho-ATM (Ser1981) (D6H9), anti-phospho-Chk1 (Ser345) (133D3), and anti-phospho-Chk2 (Thr68) (C13C1) were from Cell Signaling Technology (Beverly, MA, USA); anti-GAPDH and anti-HA-tag were from Bioworld Technology (St. Louis Park, MN, USA.).

### Cell viability assay

MTS assay (CellTiter 96Aqueous One Solution reagent; Promega, Shanghai, China) was used to test cell viability according to previously reported^40^. Briefly, 1×10^5^/ml cells in 100 μl were treated with either vehicle or ZnPT and other agents for 48 hours. 3 hours before culture termination, 20 μl MTS was added to the wells. The absorbance density was read on a 96-well plate reader at wavelength 490 nm. IC_50_ values were calculated.

### Cell death assay

Apoptosis was determined by flow cytometry using AnnexinV-fluoroisothiocyanate (FITC) / propidium iodide (PI) double staining. Cells were incubated with ZnPT, then collected and washed with binding buffer, then incubated in working solution (100 μl binding buffer with 0.3 μl Annexin V-FITC) for 15 min in dark. Cells were washed and resuspended with binding buffer. PI was added just before flow cytometric analysis. Annexin V/PI staining was also performed as described but *in situ*. The double stained cells were also imaged with an inverted fluorescence microscopy equipped with a digital camera (AxioObsever Z1, Zeiss, Germany). To monitor temporal changes in the incidence of cell death in the live culture condition, PI was added to the cell culture medium, and at the desired sequential time points, the cells in the culture dish were imaged with an inverted fluorescence microscopy.

### Peptidase activity assay

Fluorogenic Suc-LLVY-AMC substrate was used to assess for chymotrypsin-like activity of the proteasome [33]. To assay for *in vivo* proteasome inhibition, cancer cells were treated with ZnPT or bortezomib for 4 hours. The cells were lysed in ice-cold lysis buffer. Equal amounts of protein from each sample were then incubated at 37°C with 50 μM fluorogenic substrate. To assay for direct inhibition of the 20S proteasome *in vitro*, purified human 20S proteasomes were incubated with the agent to be tested for 60 minutes at 37°C before the addition of the fluorogenic substrate. Fluorescence intensity was measured using a spectrophotometer at excitation of 350 nm and emission of 438 nm (Varioskan Flash 3001, Thermo, Waltham, MA, USA).

### Ubiquitin chain disassembly

*In vitro* disassembly of purified polyubiquitin chains (K48-linked) was performed as described earlier. Purified 26S proteasomes (25 nM) were pre-incubated with either vehicle or ZnPT for 10 min *in vitro*, and then K48-linked chains (500 ng) were added into the reaction DUB buffer for 30 min at 37°C. The extent of chain disassembly was assessed by western blot.

### Active-site-directed labeling assays

Purified 26S proteasomes (25 nM) were dissolved in DUB buffer (25 mM Tris-HCl pH 7.4, 5 mM MgCl_2_, 20 mM NaCl, 200 μM ATP), and then treated with ZnPT (5, 50 μM) for 10 minutes before they were incubated with HA-UbVS for 1 h at 37 °C, followed by boiling in the reducing sample buffer and fractionated with SDS-PAGE. After transferred to PVDF membranes, HA-UbVS labeled DUBs were immunodetected using an HA antibody.

### UV absorption spectra

The UV absorption spectra of fixed amounts of DNA in Tris–HCl buffer solution were measured with addition of different concentrations of ZnPT. The wavelength range of the system was from 200 to 600 nm. The blanks corresponding to the buffer were subtracted to correct the absorbance at room temperature.

### Fluorescence spectra

In order to determine the optimal molar ratio of ethidium bromide (EB) to DNA, the fluorescence spectra of a fixed concentration (8.25 μM) of EB were measured with varying the concentrations of DNA from 0 to 57.75 μM. Then a 3.0 mL solution, containing a certain concentration of EB–DNA ([DNA]/[EB] = 7) complex solution, was added to a 1.0 cm quartz cuvette and titrated by successive addition of ZnPT. These solutions were allowed to stand for 5 min to equilibrate. The fluorescence spectra were measured at three different temperatures (300, 305 and 310 K) in the wavelength range of 550–800 nm, with the excitation wavelength at 526 nm. The appropriate blanks corresponding to the buffer solution were subtracted to correct the background.

### Western blot analysis

Whole cell lysates were prepared in RIPA buffer (1×PBS, 1% NP-40, 0.5% sodium deoxycholate, 0.1% SDS) supplemented with 10 mM β-glycerophosphate, 1 mM sodium orthovanadate, 10 mMNaF, 1 mM phenylmethylsulfonyl fluoride (PMSF), and 1× Roche Complete Mini Protease Inhibitor Cocktail (Roche, Indianapolis, IN, USA). Western blot analysis was performed as previously described [34]. In brief, equal amounts of total protein extracts from cultured cells were fractionated by 12% SDS-PAGE and electrically transferred onto polyvinylidenedifluoride (PVDF) membranes. Primary antibodies and appropriate horseradish peroxidase-conjugated secondary antibodies were used to detect the designated proteins. The bounded secondary antibodies on the PVDF membrane were reacted to the ECL detection reagents (Santa Cruz, CA) and exposed to X-ray films (Kodak, Rochester, NY, USA).

### Immunofluorescence staining

Immunofluorescence staining was performed as reported [35]. Briefly, cell culture was performed on cover-slips in six-well plates and allowed to grow overnight. After ZnPT or CDDP treatment, the cells were washed with PBS, fixed on ice for 5 min with 4% paraformaldehyde, washed again in 0.2% Triton X100/PBS for 3 × 5 min and permeabilized with 1% TritonX100/PBS for 5 min. Cells were blocked with 5% bovine serum albumin (BSA)/PBS for 1 h and subsequently exposed with primary antibodies, followed by incubation with FITC-conjugated goat anti-rabbit IgG antibody (Pierce Biotechnology). Cell nuclei were counterstained with DAPI for 10 min. Samples were analyzed using a confocal laser microscope (Zeiss LSM510 Meta, Germany). All images were acquired under identical settings with LSM Image browser software.

### Immunohistochemical staining

Formalin-fixed xenografts were embedded in paraffin and sectioned according to standard techniques as we previously reported [36]. Tumor xenograft sections (4 μm) were immunostained using the MaxVision kit (MaixinBiol) according to the manufacturer's instructions. The primary antibodies were used as indicated. 50 μl MaxVisionTM reagent was applied to each slide. Color was developed with 0.05% diaminobenzidine and 0.03% H_2_O_2_ in 50 mM Tris-HCl (pH 7.6), and the slides were counterstained with hematoxylin. A negative control for every antibody was also included for each xenograft specimen by substituting the primary antibody with preimmune rabbit serum.

### DUB activity assay

This was performed as reported [37]. Briefly, cell lysate (5 μg) or 26S proteasomes (25 nM) were dissolved in ice-cold DUB buffer containing 50 mM Tris-HCl (pH 7.4), 20 mM NaCl, 5 mM MgCl_2_ and 200 μM ATP, and pretreated with ZnPT or NEM for 15 minutes, then incubated with Ub-AMC substrate in a 100 μl reaction volume at 25°C. Free AMC generated from substrate cleavage was temporally recorded with a microplate reader (Varioskan Flash 3001, Thermo, USA).

### Blood samples and isolation of peripheral monocytes

Blood Monocytes Peripheral blood samples of normal controls were obtained from Guangzhou Blood Center and peripheral bone marrow samples of AML patients were obtained from discarded material utilized for routine laboratory tests at the Department of Hematology, Guangzhou First Municipal People's Hospital of Guangzhou Medical University; The use of these materials is approved by the Ethics Committee of these two Institutions with the permission of the patients and volunteers. Totally six patients with AML and six volunteers were recruited in this preclinical study. Mononuclear cells were isolated by Ficoll-Paque (Pharmacia, Uppsala, Sweden) density gradient. Mononuclear cell fraction was cultured in RPMI 1640 culture medium with 15% FBS.

### Nude mouse xenograft model

All animal protocols used were approved by the Institutional Animal Care and Use Committee of Guangzhou Medical University. The mice were obtained from Guangdong Laboratory Animal Monitoring Institute (SCXK2008-2002). The nude BALB/c mice (male, 18-22g) were housed in barrier facilities with a 12 h light dark cycle, with food and water available *ad libitum*. 1-10×10^6^ of K562 or A549 cells was inoculated subcutaneously on the flanks of 5-week-old male nude mice. After 72 h of inoculation, mice were treated with either vehicle (10% DMSO, 30% polythylene glycol 400 and 60% 0.9%NaCl) or ZnPT (5 mg/kg/day) for totally 15 days (7 intervals), respectively. Tumor volumes were recorded and calculated as previously reported [34].

### Computational modeling

To predict the binding of ZnPT toward the DUBs (USP14 and UCHL5), molecular docking studies were performed with CDOCKER protocol of Discovery Studio 2.0 [Accelrys Software Inc. (2007)]. The crystallographic structures of USP14 and UCHL5 were directly downloaded from the Protein Data Bank (PDB IDs: 2AYO and 3RIS). After removing irrelevant components, hydrogen atoms were added and their positions were minimized with a 0.01 kcal/mol / Å root mean square gradient by using the all-atom CHARMmforcefield and the Adopted Basis Newton-Raphson (NR) Algorithm. In addition, taking into account the possible hydrolysis of compound ZnPT (L1) in certain physiological conditions, the hydrolysate (L2) was selected as the docking ligand. The geometry structure of compound L2 was optimized using the DFT calculations at the B3LYP/LANL2DZ level to obtain NPA charges by using the Gaussian 03 [Revision D.01, Gaussian, Inc., Wallingford CT (2004)]. During the whole docking process, the two proteins were rigid, while the ligand L2 was flexible. The Input Site Spheres of 12 Å radius were centered on each active pocket of USP14 and UCHL5, with (x, y, z) = (38.12, 84.32, 6.61) and (-9.40, 6.57, 61.54), respectively. The conformation corresponding to the lowest CDOCKER Interaction Energy was selected as the most probable binding conformation. All parameters used in calculation were default except for explained.

### Statistical analysis

All the results were expressed as Mean ± SD where applicable. GraphPad Prism 4.0 software (GraphPad Software) was used for statistical analysis. Student's t test was used to compare the differences between variables. P value of <0.05 was considered statistically significant.

This work was supported in part by the National High Technology Research and Development Program of China (2006AA02Z4B5); NSFC (81272451/H1609, 81472762/H1609) Key Projects (10A057S) from Guangzhou Education Commission and a project (2010A060801016) from Guangdong Key Laboratory of Urology (to J.L.); NSFC (81201719/H1609) (to H.H.) and US NIH grants HL072166 and HL085629 (to X.W.).
